# Combined administration of gallic acid and glibenclamide mitigate systemic complication and histological changes in the cornea of diabetic rats induced with streptozotocin

**DOI:** 10.1590/acb390124

**Published:** 2024-02-05

**Authors:** Jing Zhao, Shaik Althaf Hussain, Narendra Maddu

**Affiliations:** 1 Sanmenxia Central Hospital – Department of Ophthalmology – Sanmenxia – China.; 2 King Saud University – College of Science – Department of Zoology – Riyadh – Saudi Arabia.; 3 Sri Krishnadevaraya University – Department of Biochemistry – Anantapur – India.

**Keywords:** Hyperglycemia, Antioxidants, Diabetes Complications

## Abstract

**Purpose:**

To determine the effect of gallic acid or its combination with glibenclamide on some biochemical markers and histology of the cornea of streptozotocin (STZ) induced diabetic rats.

**Methods:**

Following induction of diabetes, 24 male albino rats were divided into four groups of six rats each. Groups 1 and 2 (control and diabetic) received rat pellets and distilled water; group 3 (gallic acid) received rat pellets and gallic acid (10 mg/kg, orally) dissolved in the distilled water; and group 4 (gallic acid + glibenclamide) received rat pellets, gallic acid (10 mg/kg, orally), and glibenclamide (5 mg/kg, orally) dissolved in the distilled water. The treatments were administered for three months after which the rats were sacrificed after an overnight fast. Blood and sera were collected for the determination of biochemical parameters, while their eyes were excised for histology.

**Results:**

STZ administration to the rats induced insulin resistance, hyperglycemia, microprotenuria, loss of weight, oxidative stress, inflammation, and alteration of their cornea histology, which was abolished following supplementation with gallic acid or its combination with glibenclamide.

**Conclusions:**

The study showed the potentials of gallic acid and glibenclamide in mitigating systemic complication and histological changes in the cornea of diabetic rats induced with STZ.

## Introduction

Diabetes mellitus (DM) is a group of metabolic disorders that affect the metabolism of carbohydrates, lipids, and proteins^1^. The increasing global prevalence of DM and its association comorbidities has made DM one of the diseases of great concern in the XXI century. With the increasing prevalence of DM, ocular complications, which are among the comorbidities of DM, have come to light as key causes of blindness worldwide^2^. Studies have shown that over 25% of people that suffer from DM have ocular complications, such as: retinopathy, glaucoma, cataract, scotoma, and corneal abnormalities (collectively called diabetic keratopathy)^3^^–^ 7.

Whereas many studies have focused on diabetic retinopathy (DR), few studies investigated diabetic cornea complications^2^. Research in this area has become necessary since corneal complication was reported as the third leading cause of blindness that provokes serious damage to the visual quality of patients’ life^8^^,^ 9, and epidemiological studies reported that about 70% of diabetic patients suffer from cornea complications/pathology during the process of DM^7^^,^ 10.

One of the key culprits in the pathogenesis of ocular complications is oxidative stress^11^. The eye is more susceptible to oxidative stress than other body tissues because of its continuous exposure to environmental chemicals, radiation and atmospheric oxygen, its high levels of membrane bound polyunsaturated fatty acids, higher glucose oxidation, and oxygen utilization. Oxidative stress induced by hyperglycemia triggers mitochondrial dysfunction and reactive oxygen species (ROS) accumulation, depleting the antioxidant defense systems. This leads to a decline in corneal epithelial cell density and loss of epithelial function, which are involved in the pathogenesis of diabetic corneal complications^2^^,^ 9 ^,^ 11.

Chronic hyperglycemia also causes increased expression of proinflammatory mediators such as cytokines and chemokines, as well as over expression of proapoptotic genes, that lead to the initiation and progression of diabetic ocular diseases including corneal complication^2^^,^ 12. In fact, inflammatory mediators such as tumor necrosis factor-a (TNF-a), interleukins, and vascular endothelial growth factor (VEGF) can cause morphological changes in the cornea, leading to decreased functionality of the cornea^2^^,^ 12. Although the exact way hyperglycemia causes blindness has not been fully understood, several mechanisms have been proposed, such as systemic and intraocular formation of ROS and pro-inflammatory cytokines that lead to pathological and biochemical changes, for example protein glycation (as seen in HbA1C) and advanced glycation end products (AGEs) formation, increased VEGF expression and cellular signaling by vascular basement membrane thickening^13^^–^ 17.

While several interventions–metformin, glibenclamide, acarbose etc.–are currently being used to manage DM, these drugs have not been found to be very effective in preventing the progression to diabetic complications or in treating these diabetic complications. In addition, their usage has also been associated with adverse effects. Therefore, alternative approaches that can be used to manage DM and prevent its progression to the development of complications including ocular complications are currently being sought after. The use of supplements and plant compounds to enhance the body’s antioxidant system can prevent over production of ROS and the development of diabetes and related complications including onset of ocular complications^11^ that can progress to loss of vision.

Gallic acid (GA) is a plant polyphenol that is found in vegetables, grapes, berries, tea, fruit juices, and wine. Some of the pharmacological properties credited to gallic acid include antioxidant, anti-obesity, anti-inflammatory, antimutagenic, cardioprotective, antimicrobial, anticancer, and antihyperglycemic properties^18^^,^ 19.

Glibenclamide (GBL) is a second-generation sulfonylurea that achieves glycemic control by provoking insulin release through blockage of the ATP-sensitive K^+^ channels and depolarization of the pancreatic beta cells^20^^,^ 21. The drug is reported to be effective at decreasing plasma glucose concentration and reducing HbA1C by approximately 1 to 2%^22^. Further, the capacity of GBL to mitigate oxidative stress biomarkers in experimental models of DM has been reported^21^.

In recent decades, there has been a heightened interest in the use of combined therapy in treating different medical conditions^21^. This approach has especially been recommended for DM treatment to improve treatment outcomes, maintain a normal physiological level of HbA1C, prevent the development of microvascular and macrovascular complications and mitigate potential adverse effects^21^^,^ 23 ^,^ 24. Whereas GA and GBL monotherapies have been reported in the management of DM in experimental animals^18^^–^ 21, the effect of the combined therapy on diabetes management in humans or experimental animals has not been reported.

Considering the increasing global prevalence of DM and its contribution to ocular complications globally, and the limitations of the currently used antidiabetic drugs in preventing progression to diabetic complications, this study was designed with the following objectives:

- To determine the possibility of achieving better glycemic control (using blood glucose and glycated hemoglobin-HbA1C as markers) with GA and GBL polytherapy compared with GA alone in streptozotocin (STZ) induced diabetic rats;
- To evaluate the effect of the polytherapy on the body weights, serum insulin, homeostatic model assessment insulin resistance (HOMA-IR), oxidative stress index (malondialdehyde–MDA), antioxidant status (superoxide dismutase–SOD, glutathione peroxidase–GPx, reduced glutathione–GSH, glutathione-S-transferase–GST), oxidative stress index (MDA) and inflammatory markers (nuclear factor kappa B–NFkB, TNF-α, inducible nitric oxide synthase–iNOS, VEGF) in the sera of the diabetic rats;
- To determine the effect of the combined therapy on the histological changes in the cornea of the diabetic rats.

## Methods

### Chemicals

The enzyme-linked immunosorbent assay (ELISA) kits used for this study were products of Elab Science, China. Other used chemicals/reagents not itemized herein were also of the highest analytical quality.

### Animals

Thirty male albino Wistar rats (aged 6 weeks, with weight of 130 to 150 g) were purchased for this study. The rats were kept singly in stainless cages under a 12-h light/dark cycle and temperature of 22°C ± 2°C and were allowed to acclimate for two weeks to their diets and environment. Ethical approval for this study was granted by the Sanmenxia Central Hospital Animal Ethical Committee (research ethics approval number 211B01). All the guidelines for the care and handling of laboratory animals, as reported by the US National Institute of Health, were followed.

### Induction of diabetes mellitus

After the acclimation period, STZ solution was prepared by dissolving 0.1 g of STZ in 5 mL of freshly prepared sodium citrate buffer 0.1 M, pH 4.5. The solution was immediately injected to 24 of the rats, intraperitoneally at the dose of 65 mg/kg body weight, following an overnight fast, while the remaining six rats served as the control. Five percent glucose water was provided *ad libitum* to the rats, and it was removed the next day to partially protect the pancreatic beta cells of the rats from STZ induced hypoglycemia and prevent early mortalities.

Type 1 DM was confirmed seven days after STZ injection with a fasting blood glucose concentration = 200 mg/dL using a blood glucose meter. Eighteen rats were confirmed to be diabetic, while the rest of the STZ administered rats were excluded from the study.

### Experimental design

The rats (control and diabetic) were categorized into four groups of six rats each and treated as follows:

- Group 1 (control): received rat pellets and distilled water;
- Group 2 (diabetic): received rat pellets and distilled water;
- Group 3 (GA): received rat pellets and oral administration GA (10 mg/kg) dissolved in the distilled water;
- Group 4 (GA + GBL): received rat pellets, GA (10 mg/kg, orally) and GBL (5 mg/kg, orally) dissolved in the distilled water.

The doses of GA and GBL that were chosen for this study were based on previous studies^19^^,^ 21. The body weights of the rats were measured weekly, while their feed intakes were measured daily. The treatments or diets were administered for three months after which the rats were fasted overnight, and, on the next day, their blood glucose concentrations were determined with a glucose meter, using the blood that was obtained by pricking their tails. Their final body weights were also recorded. The rats were subsequently anesthetized with ketamine (90 mg/kg) and xylazine (5 mg/kg), and blood was drawn from their heart.

The harvested blood samples were divided into two parts. The first part was kept in plain tubes and centrifuged (3,000 × g for 10 min) to obtain the sera, which were analyzed for insulin, total protein, albumin, SOD, GPx, GSH, GST, MDA, NFkB, TNF-a, iNOs, and VEGF concentrations. The second part of the blood was put in heparin tubes for the analysis of HbA1C.

### Preparation of the eyes for histology

The right eyes of the rats were collected and blotted. They were subsequently fixed in 10% formalin fixative and used for histology studies. The fixed eye tissues were sliced to 1 cm thickness and put in cassettes. Subsequently, they were processed, embedded in molten paraffin, and cooled to form paraffin blocks. The blocks were sectioned at 5 μm and stained with hematoxylin and eosin (H&E) for viewing under a light microscope. Pathological pictures of the cornea were taken at × 400 under an optical microscope (Olympus BX51, Japan).

### Determination of serum insulin concentration, insulin resistance, and blood HbA1C

Insulin assay was conducted with assay kits for insulin (Monobind Inc.), and results were reported as µU/mL. Homeostasis model assessment of insulin resistance (HOMA-IR) was calculated using Eq. 1^25^:


HOMA-IR=[Serum insulin(μU/mL)×fasting blood glucose(mmol/L)/22.5) (1)

Assay for HbA1C concentrations in the rats’ blood was done following Karl et al.’s^26^ procedure.

### Determination of serum total proteins and albumin in the sera

The concentrations of total proteins and albumin in the sera of the rats were determined using their respective kits (Biosystems kits) following the manufacturer’s described protocol.

### Assay of antioxidant status and lipid peroxidation in the sera

The SOD activity in the rats’ sera was measured using Winterbourn et al.’s^27^ method. Results were reported as units per mL. GPx activity in the sera of the rats was measured following Dogan et al.’s^28^ method, and results were reported as units per mL. The concentration of reduced glutathione (GSH) was analyzed using Annuk et al.’s^29^ method, and results were reported as µmol/mL. GST activity was measured using Habig et al.’s^30^ method, and results were reported as units/mL. Lipid peroxidation in the rats’ sera was analyzed following Ohkawa et al.’s^31^ protocol, and results were expressed as nmol of MDA/mL.

### Assay of pro-inflammatory cytokines in the sera

The concentrations of the cytokines-NFkB, TNF-a, iNOs and VEGF in the sera of the rats were measured using ELISA kits (rats’ NF-kB ELISA kit: CUSABIO; rats’ TNF-a ELISA kit: Elabscience; rats’ iNOS ELISA kit: CUSABIO; and rats’ VEGF ELISA kit: CUSABIO), following the protocols that were described by the manufacturer. Results obtained were expressed as pg per mL for NFkB and TNF-a, units/mL for iNOS and pg/mL for VEGF, respectively.

### Statistical analysis

Statistical analyses were performed using the IBM Statistical Package for the Social Sciences 23 software (SPSS, Chicago, IL, United States of America). Data were reported as mean ± standard error. One-way analysis of variance (ANOVA) test was performed, and post hoc multiple comparisons were conducted using Duncan’s multiple range test. Statistical significance was set at p < 0.05. Graphs were generated using GraphPad Prism version 9.5.1 (GraphPad Software Inc., San Diego, CA, United States of America).

## Results

### Blood glucose concentrations of rats

**Figure 1:**
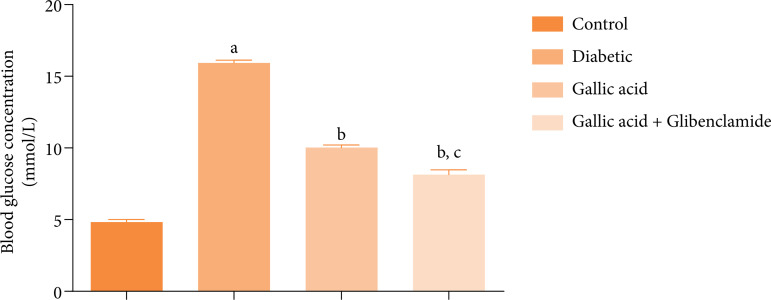
Effect of gallic acid and glibenclamide on the blood glucose concentrations of streptozotocin diabetic rats.

Figure 1 presents the effect of GA or GA and GBL on the blood glucose concentrations of STZ induced diabetic rats. Data presented in Fig. 1 showed significant increases (P < 0.05) in the blood glucose concentrations of the diabetic rats compared with the control. Supplementation with GA or GA and GBL significantly decreased the glucose concentrations of the rats relative to the diabetic group. Further, the blood glucose concentrations of the rats supplemented with GA and GBL were decreased significantly (P < 0.05) when compared to the blood glucose concentrations of the rats that received only GA.

Data are reported as means ± standard error; ^a^P < 0.05 in comparison with the control; ^b^P < 0.05 in comparison with the diabetic group; ^c^P < 0.05 in comparison with the gallic acid group; N = 6 rats per group. Source: elaborated by the authors.

### Blood HbA1C, serum insulin concentrations, and insulin resistance

**Figure 2:**
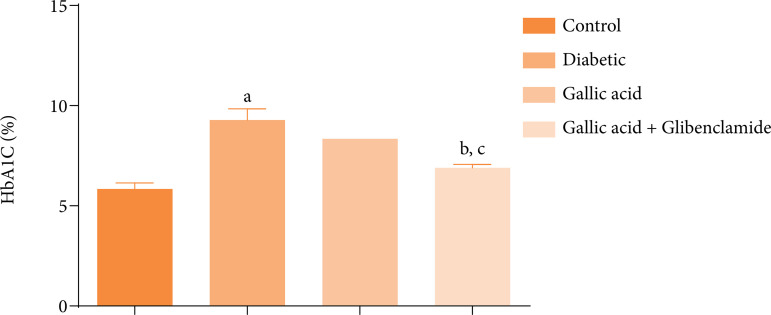
Effect of gallic acid and glibenclamide on the glycated hemoglobin (HbA1C) concentrations in the blood of streptozotocin diabetic rats.

Figure 2 presents the effect of GA or GA and GBL on the blood HbA1C concentrations of diabetic rats induced with STZ. Significant increases (P < 0.05) in HbA1C concentrations were obtained for the diabetic rats compared with the control. Whereas supplementation with GA did not significantly decrease (P > 0.05) the HbA1C concentrations of the rats relative to the diabetic group, combined administration of GA and GBL significantly decreased (P < 0.05) the HbA1C concentrations of the rats when compared to the diabetic group. Further, the HbA1C concentration of the rats supplemented with GA and GBL decreased significantly (P < 0.05) when compared to the HbA1C concentrations of the rats that received only GA.

Data are reported as means ± standard error; ^a^P < 0.05 in comparison with the control; ^b^P < 0.05 in comparison with the diabetic group; ^c^P < 0.05 in comparison with the gallic acid group; N = 6 rats per group. Source: elaborated by the authors.

**Figure 3:**
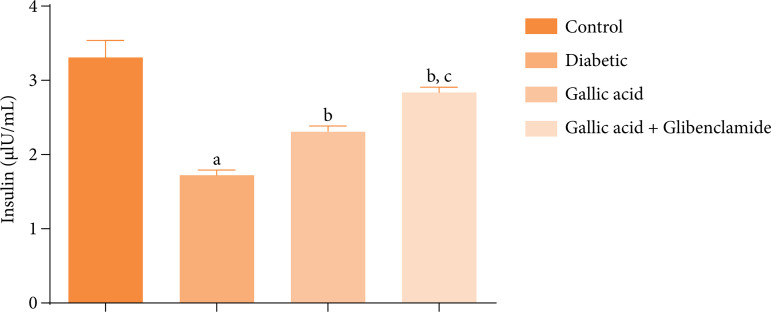
Effect of gallic acid and glibenclamide on the serum insulin concentrations in streptozotocin diabetic rats.

Figure 3 shows the effect of GA and GBL on the serum insulin concentrations of STZ diabetic rats. Significant decreases (P < 0.05) were obtained in the insulin concentrations of the diabetic group compared to the control. However, supplementation with GA or GA and GBL significantly increased (P < 0.05) the insulin concentrations of the rats with respect to the diabetic group. Additionally, the serum insulin concentrations of the diabetic rats administered GA and GBL were significantly higher (P < 0.05) than the serum insulin concentrations of the diabetic rats that received only GA.

Data are reported as means ± standard error; ^a^P < 0.05 in comparison with the control; ^b^P < 0.05 in comparison with the diabetic group; ^c^P < 0.05 in comparison with the gallic acid group; N = 6 rats per group. Source: elaborated by the authors.

**Figure 4:**
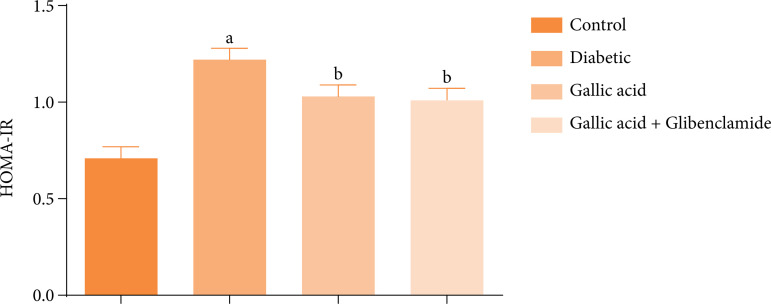
Effect of gallic acid and glibenclamide on the homeostatic model assessment of insulin resistance (HOMA-IR) in streptozotocin diabetic rats.

Figure 4 shows the effect of GA and GBL on the HOMA-IR in diabetic rats induced with STZ diabetic. Significant increases (P < 0.05) were obtained for the HOMA-IR values in the diabetic group relative to the control. On the contrary, the HOMA-IR values of the diabetic rats administered GA or GA and GBL polytherapy were significantly decreased (P < 0.05) relative to the diabetic group.

Data are reported as means ± standard error; ^a^P < 0.05 in comparison with the control; ^b^P < 0.05 in comparison with the diabetic group; ^c^P < 0.05 in comparison with the gallic acid group; N = 6 rats per group. Source: elaborated by the authors.

### Body weights of rats

Table 1 shows the effect of GA and GBL on the body weights of STZ diabetic rats. The rats in the control, diabetic, GA, and GA + GBL groups had statistically similar initial body weights when the study started. At the end of the study, the final body weights of the diabetic group were significantly decreased (P < 0.05) with respect to the control group. In contrast, the final body weights of the rats in the GA or GA and GBL groups were significantly increased (P < 0.05) when compared to the diabetic group.

**Table 1:** Effect of gallic acid and glibenclamide on the initial and final body weights of streptozotocin diabetic rats.

Groups	Initial weight (g)	Final weight (g)
Control	147.05 ± 2.20	294.48 ± 3.78
Diabetic	151.30 ± 2.73	232.7 ± 1.17^a^
Gallic acid	143.42 ± 3.74	246.50 ± 1.98^b^
Gallic acid + glibenclamide	145.82 ± 2.23	252.31 ± 3.53^b^

Values in the table are given as means ± standard error;

a P < 0.05 in comparison with the control;

b P < 0.05 in comparison with the diabetic group;

N = 6 rats per group.

Source: elaborated by the authors.

### Total proteins and albumin in the sera of rats

Table 2 shows the effect of GA and GBL on the serum total proteins and albumin concentrations in STZ diabetic rats. The total proteins and albumin concentrations in the sera of the diabetic group were significantly decreased (P < 0.05) with respect to the control group. Conversely, supplementation with GA or GA and GBL significantly increased (P < 0.05) the total proteins and albumin concentration in the sera of the rats with respect to the diabetic group.

**Table 2:** Effect of gallic acid and glibenclamide on the total proteins and albumin concentrations in the sera of streptozotocin diabetic rats.

Groups	Total protein (g/dL)	Albumin (g/dL)
Control	7.69 ± 0.26	4.17 ± 0.17
Diabetic	4.73 ± 0.14^a^	2.01 ± 0.15^a^
Gallic acid	5.52 ± 0.25^b^	3.08 ± 0.28^b^
Gallic acid + glibenclamide	6.10 ± 0.14^b^	3.38 ± 0.26^b^

Values in the table are given as means ± standard error;

a P < 0.05 in comparison with the control;

b P < 0.05 in comparison with the diabetic group;

N = 6 rats per group.

Source: elaborated by the authors.

### Oxidative stress marker and antioxidant status in the sera

Table 3 shows the effect of GA and GBL on the oxidative stress and antioxidant status in the sera of diabetic rats induced with STZ. Significant increases (P < 0.05) were obtained for the serum MDA concentrations of the diabetic rats compared to the control group. Supplementation with GA or GA and GBL significantly decreased (P < 0.05) the MDA concentrations in the sera of the rats when compared to the diabetic group.

**Table 3:** Effect of gallic acid and glibenclamide on the oxidative stress and antioxidant status in the sera of streptozotocin diabetic rats.

Groups	Malondialdehyde (µmol/mL)	Superoxide dismutase (Units/mL)	Glutathione peroxidase (Units/mL)	Reduced glutathione (µmol/mL)	Glutathione-S-transferase (Units/mL)
Control	0.40 ± 0.05	6.67 ± 0.42	52.55 ± 2.97	3.89 ± 0.25	192.50 ± 6.49
Diabetic	0.87 ± 0.03^a^	3.41 ± 0.39^a^	30.62 ± 3.11^a^	1.79 ± 0.15^a^	185.33 ± 7.37
Gallic acid	0.65 ± 0.02^b^	4.91 ± 0.27^b^	38.60 ± 1.64^b^	2.64 ± 0.33^b^	190.17 ± 6.56
Gallic acid/ glibenclamide	0.59 ± 0.04^b^	5.27 ± 0.51^b^	40.65 ± 2.58^b^	2.86 ± 0.23^b^	187.83 ± 6.38

Data in the table are reported as means ± standard error; N = 6 rats per group. Source: elaborated by the authors.

a P < 0.05 in comparison with the control;

b P < 0.05 in comparison with the diabetic group;

N = 6 rats per group.

Source: elaborated by the authors.

The antioxidant markers-SOD, GPx and GSH were significantly decreased (P < 0.05) in the sera of the diabetic group compared to the control. Supplementation with GA or GA and GBL significantly increased (P < 0.05) the serum activities/concentrations of SOD, GPx and GSH in the rats with respective to the diabetic group.

The serum GST activities of the diabetic rats were not significantly different (P > 0.05) from the control. Supplementation with GA or GA and GBL did not produce any significant change (P > 0.05) in the activities GST in the sera of the rats when compared to the diabetic group.

### Pro-inflammatory cytokines in the sera

The effect of GA or GA and GBL on the expressions of pro-inflammatory cytokines in the sera of diabetic rats induced with STZ is presented in Table 4. Significant increases (P < 0.05) in the serum expressions of NFkB, TNF-a and iNOS were obtained in the diabetic rats with respect to the control. Supplementation with GA or GA and GBL significantly decreased (P < 0.05) the serum expressions of NFkB, TNF-a and iNOS in the rats when compared to the diabetic group.

**Table 4:** Effect of gallic acid and glibenclamide on the expressions of pro-inflammatory markers in the sera of streptozotocin diabetic rats.

Groups	Nuclear factor kappa B (pg/mL)	Tumor necrosis factor alpha (pg/mL)	Inducible nitric oxide synthase (units/mL)
Control	346.82 ± 20.28	154.98 ± 7.20	0.92 ± 0.03
Diabetic	530.60 ± 19.79^a^	237.02 ± 19.40^a^	2.60 ± 0.35^a^
Gallic acid	448.09 ± 20.76^b^	194.54 ± 10.41^b^	1.65 ± 0.12^b^
Gallic acid + glibenclamide	413.75 ± 23.49^b^	191.64 ± 7.42^b^	1.53 ± 0.11^b^

Values in the table are reported as means ± standard error;

a P < 0.05 in comparison with the control;

b P < 0.05 in comparison with the diabetic group;

N = 6 rats per group.

Source: elaborated by the authors.

**Figure 5:**
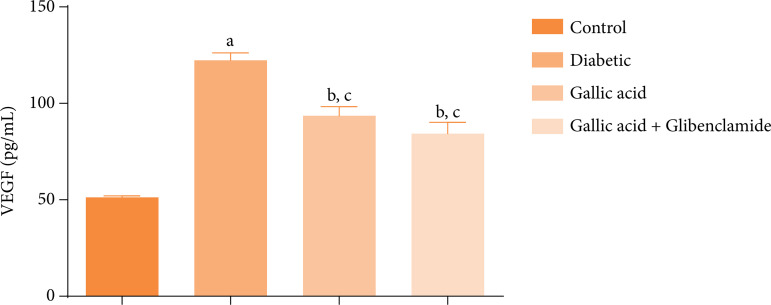
Effect of gallic acid and glibenclamide on the vascular endothelial growth factor (VEGF) in the sera of streptozotocin diabetic rats.

Figure 5 presents the effect of GA or GA and GBL on the serum expressions of VEGF in diabetic rats induced with STZ. Significant increases (P < 0.05) were obtained in the serum expression of VEGF in the diabetic rats compared with to control.

Data are reported as means ± standard error; ^a^P < 0.05 in comparison with the control; ^b^P < 0.05 in comparison with the diabetic group; ^c^P < 0.05 in comparison with the gallic acid group; N = 6 rats per group. Source: elaborated by the authors.

Supplementation with GA or GA and GBL triggered significant decreases (P<0.05) in the expression of VEGF in the sera of the rats compared to the diabetic group. In addition, there were also significant increases (P < 0.05) in the serum VEGF concentrations of the diabetic rats administered GA or GA and GBL compared to the control.

### Histology results

**Figure 6:**
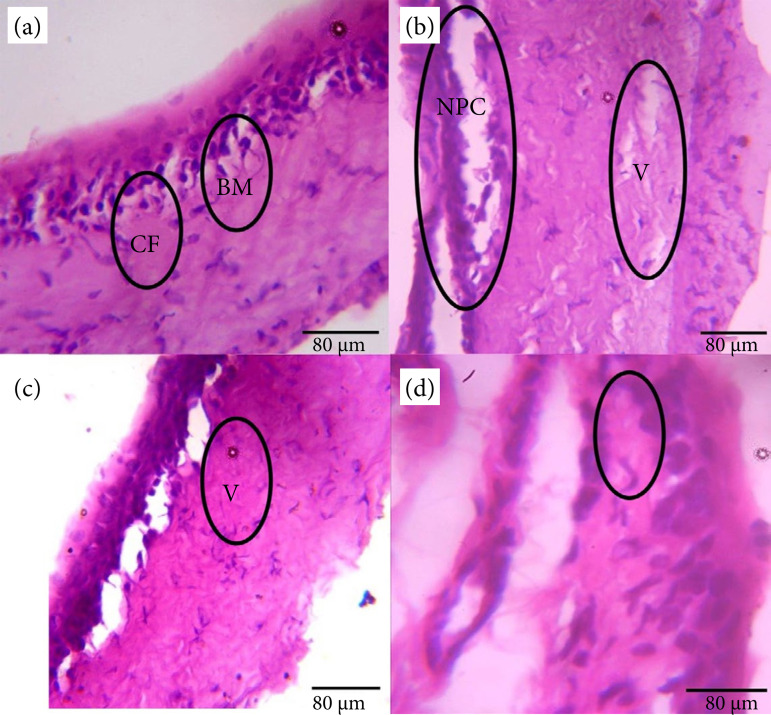
Effect of gallic acid and glibenclamide on the histology of the cornea of streptozotocin diabetic rats. **(a)** Histology of the cornea of the control (hematoxylin & eosin) (× 400) shows a multillayered structure displaying acellular bowmans membrane (BM) with collagen fibre (CF) and intervening fibroblast. **(b)** Histology of the cornea of the eye of the diabetic group (hematoxylin & eosin) (× 400) shows severe cornea edema with severe necrotic epithelia cells (NPC) and severe vascularization of acellular Bowmans membrane **(V)** and infiltration of inflammatory cells (IC). **(c)** Histology of the cornea of the eye of the gallic acid group (hematoxylin & eosin) (× 400) shows mild regeneration with mild vasclarization **(V)** of the acellular bowmans membrane. **(d)** Histology of the cornea of the eye of the gallic + glibenclamide group (hematoxylin & eosin) (× 400) shows evidence of regeneration with active appearance of the epithelial cells and fibroblast (encircled areas), and except very mild vascularization **(V)** of the acellular bowmans membrane, its otherwise normal.

Source: Elaborated by the authors

Figure 6 shows the effect of GA or GA and GBL on the cornea histology of STZ induced diabetic rats.

## Discussion

In this study, STZ administration to the diabetic group induced significant and chronic hyperglycemia in the rats, as evident from the elevation in their circulating glucose and HbA1C concentrations.

It has been reported that chronic exposure to hyperglycemia favors the nonenzymatic glycation of proteins (hemoglobin, albumin, and low-density lipoprotein), resulting in the formation of early and advanced glycation end products (AGEs)^14^^,^ 32 ^,^ 33. These glycation end products have been proposed to be responsible for corneal and other ocular complications arising from uncontrolled DM^34^. HbA1C is an early glycation end product, and its assay is used to diagnose prolonged diabetes and to assess control of glycemia in diabetic individuals^35^^,^ 36. In addition, it has been reported that DM with increased HbA1C concentrations predisposes the cornea to epithelial barrier dysfunction, leading to corneal complication^37^.

The study showed that GBL potentiated the anti-hyperglycemic properties of GA, as evident from the higher blood glucose lowering action of combination of GA and GBL compared with GA alone, as obtained in this study. Further, while supplementation with GA did not decrease the elevated HbA1C concentrations of the rats, combination of GA and GBL abrogated the elevated HbA1C concentrations of the rats, indicating better glycemic control following combination of GA and GA unlike GA monotherapy. It is likely that the duration of treatment with GA alone was not enough to obtain a significant reduction of HbA1C in the diabetic rats, unlike combination of GA and GBL, affirming previous reports on the benefits of combined therapy in the treatment of DM^21^^,^ 23 ^,^ 24.

In this study, decreased serum insulin concentrations and increased insulin resistance index (HOMA-IR) values were obtained in the diabetic rats. It is known that STZ diabetes model leads to impairment of the ß-cells of the pancreas, leading to decreased synthesis and release of insulin into circulation. Additionally, it has been established that insulin resistance and decreased insulin secretion are key parameters that contribute to the onset of DM^36^. These might therefore explain the decreased circulating insulin and increased HOMA-IR values in the diabetic group^38^.

The study further showed that the decreased serum insulin concentration and increased HOMA-IR values in the diabetic rats were reversed following supplementation with GA. Similar reports on the modulation of circulating insulin and HOMA-IR values by GA were given by Gandhi et al.^39^ on diabetic rats. In addition, combined administration of GA and GBL further potentiated the insulin secreting action of GA and also decreased the HOMA-IR value in the diabetic rats, suggesting a synergy of interaction between GA and GBL at the doses used in this study. Indeed, GBL is a sulfonylurea that abates hyperglycemia by stimulating the release of insulin from the pancreatic ß-cells^40^. The study suggests stimulation of insulin secretion and sensitivity as a possible mechanism of the antidiabetic action of GA and GBL polytherapy, as obtained in this study.

The current study further showed significant weight loss in the diabetic group, which was mitigated following supplementation with GA or combination of GA and GBL. The loss of weight by the diabetic rats could be attributed to increased muscle wasting due to catabolism of structural proteins (in the absence of insulin) to make up for the unavailability of carbohydrates that are used as energy source^41^. On the contrary, the gain in weight in the rats administered GA or GA and GBL could be attributed to decreased protein catabolism as a result of mitigation of hyperglycemia by GA or combination of GA and GBL.

The present study showed decreased total protein and albumin concentrations in the sera of the diabetic rats, which were increased following supplementation with GA or its combination with GBL. During DM, there is increased protein catabolism with the release of amino acids as energy source (via gluconeogenesis) to make up for the unavailability of carbohydrates that can be used as energy source^41^^–^ 43. This might explain the observed decrease in the serum total protein and albumin concentrations of the diabetic group. The increased concentrations of total proteins and albumin in the sera of the rats administered GA or its combination with GBL could be attributed to attenuate hyperglycemia by GA or its combination with GBL, leading to decreased protein catabolism.

The blood of diabetic patients is continuously exposed to oxidative stress as ROS are continuously generated by auto-oxidation of hemoglobin. The generated ROS in turn attacks the polyunsaturated fatty acids of lipid membranes, leading to generation of lipid peroxides and MDA^44^, depleting the body’s antioxidant defense systems. Hyperglycemia induced alteration of intracellular antioxidant status and increased systemic oxidative stress has been reported to affect the structure and function of the cornea, leading to delayed epithelial wound healing and severe epithelial cell damage in diabetic corneas^2^^,^ 45 ^,^ 46.

The present study showed increased oxidative stress in the sera of the diabetic group as evidenced from increased serum concentrations of the lipid peroxidation product, MDA, but decreased concentrations of the antioxidant defense systems: SOD, GPx and GSH in the sera of the diabetic group. GA alone or its combination with GBL demonstrated the capacity to mitigate STZ induced systemic oxidative stress, as seen from the decreased serum concentrations of MDA, but increased antioxidant defense systems–SOD, GPx and GSH in the diabetic rats treated with GA or GA and GBL.

The non-significant change in the serum GST activities of the diabetic untreated rats and the diabetic rats administered GA or GA and GBL cannot be explained in this current study, but it is noteworthy.

Low grade chronic inflammation has been also reported as playing a contributory role to the development of diabetes and its related complications including diabetic keratopathy^47^. Although low systemic inflammation can lead to diabetic corneal complication has not been fully unbundled, it has been suggested that systemic control of DM aided by anti-inflammatory agents can improve ocular surface health^48^. NFkB is a redox transcription factor that is activated by oxidative stress. Activation of NFkB induces the release of other pro-inflammatory mediators such as TNF-a and others^48^^,^ 49. TNF-a plays a critical role in maintaining the inflammatory response and increasing ROS generation in the tissues including the cornea^2^^,^ 12 ^,^ 50 ^,^ 51. Activation of TNF-a triggers the activation of iNOS that is present in macrophages^1^. It has been reported that increased generation of these ROS and inflammatory mediators can cause oxidative damage to the cornea^32^^,^ 34 ^,^ 51.

As observed in this study, STZ induction triggered systemic inflammation in the diabetic group as evidenced from the increased expression of iNOS and the pro-inflammatory cytokines-NFkB and TNF-a in the sera of the diabetic group, which was abrogated after supplementation with GA or its combination with GBL.

The decreased serum concentrations of NFkB and TNF-a, as well as the decreased serum iNOS activity, in the diabetic rats administered GA or combination of GA and GBL suggest the anti-inflammatory properties of GA or GA and GBL polytherapy.

The pro-angiogenic molecule–VEGF–has been reported to play a significant role in neovascularization of the cornea^52^ and in promoting morphological changes in the cornea that can lead to its decreased functionality^2^^,^ 12. As seen in this study, STZ induction caused an elevation in the serum concentrations of VEGF in the diabetic rats, which was mitigated following treatment with GA or GA and GBL polytherapy.

A number of systemic antioxidant and anti-inflammatory therapies such as beta carotene (an antioxidant), Resolvin-D1 (an anti-inflammatory eicosanoid), enalapril (an ACE inhibitor) with alpha lipoic acid (an antioxidant) were reported to be beneficial in mitigating corneal degeneration in diabetic rats, when administered orally^48^^,^ 53 ^–^ 55. These studies and more support earlier reports that systemic treatment in DM is the focal point for the treatment of any diabetes associated complication including diabetic corneal complication^48^.

Corneal sections of diabetic rats in previous studies^44^^,^ 56 showed severe degenerative changes, such as edema, degradation of its collagen fibers, and corneal neovascularization, which are consistent with the findings of this study. Severe vascularization of acellular bowmans membrane, corneal edema and cornea fibrosis as obtained in the histology of the cornea of the diabetic group suggests that they were at risk of developing diabetic corneal complications. Treatment with GA or its combination with GBL significantly restored the histological features of the cornea, indicating the protective action of GA or GA and GBL polytherapy against STZ-diabetes induced cornea pathology.

To the best of our knowledge, this is the first report on the effect of combined administration of GA and GBL on hyperglycemia, systemic oxidative stress, inflammation and histological changes in the cornea of diabetic rats.

A limitation in this study is that the molecular basis for the improved histological changes in the cornea of the diabetic rats treated with GA or GA and GBL could not be determined. Additionally, we were unable to determine the oxidative stress and inflammatory markers in the cornea of the diabetic rats. Further studies to determine the impact of GA or GA and GBL on oxidative stress, and inflammatory markers in the cornea of the diabetic rats, and to understand the molecular basis of the improved corneal histological changes in the diabetic rats treated with these interventions are recommended.

## Conclusion

The study showed that GA and GBL polytherapy exerted better glycemic control than GA by itself. Further, GA or its combination with GBL demonstrated antioxidant and anti-inflammatory properties in the diabetic rats.

Finally, the study showed the potentials of GA or GA and GBL polytherapy in mitigating diabetes induced histological changes in the cornea in experimental rats.

## Data Availability

Data will be available upon request.
